# Cryonics, euthanasia, and the doctrine of double effect

**DOI:** 10.1186/s13010-023-00137-5

**Published:** 2023-06-29

**Authors:** Gabriel Andrade, Maria Campo Redondo

**Affiliations:** 1 Ajman University, Ajman, UAE; 2 United Arab Emirates University, Al Ain, UAE

**Keywords:** Cryonics, Euthanasia, Double effect, Death

## Abstract

In 1989, Thomas Donaldson requested the California courts to allow physicians to hasten his death. Donaldson had been diagnosed with brain cancer, and he desired to die in order to cryonically preserve his brain, so as to stop its further deterioration. This case elicits an important question: is this a case of euthanasia? In this article, we examine the traditional criteria of death, and contrast it with the information-theoretic criterion. If this criterion is accepted, we posit that Donaldson’s case would have been cryocide, but not euthanasia. We then examine if cryocide is an ethically feasible alternative to euthanasia. To do so, we rely on the ethical doctrine of double effect.

## Introduction: the strange case of Thomas A. Donaldson

In recent years, public support for euthanasia has increased, and consequently, legislators are at greater ease in attempts to legalize euthanasia. This has been materialized in legal reforms in Netherlands, Belgium, Luxembourg, Canada, Colombia, Germany, Switzerland, Japan, and some states in the United States [9], allowing for euthanasia and physician-assisted suicide in some circumstances.

Although there have been plenty of cases related to euthanasia that have been on the spotlight of media coverage (e.g., Dr. Jack Kevorkian’s antics, or the legal battle surrounding Terry Schiavo’s fate), the case of Thomas Donaldson did not get much media attention, but it arouses very complex legal and philosophical questions [23].

Donaldson was a 46-year-old man who in 1988 was diagnosed with a brain tumor. Physicians predicted he would die by 1993. As opposed to most other patients who request euthanasia, Donaldson’s quality of life was not exceptionally poor. Yet, Donaldson requested the California courts to grant him the right to be assisted in hastening his death. His rationale was that hastening his death would stop the deterioration of his brain (given that the tumor was growing), and this would allow for his brain to be cryonically preserved in an optimal state after his death.

As per cryonics procedures, shortly after a person is legally declared to be dead, the body (or alternatively, only the head) is preserved at -150 degrees, in the hope that at some future time, advances in biomedical technology would permit the revival of the person [21], thus hoping to preserve the original identity. As we will see, the very concept of identity may be fraught with difficulties, but this is not an unsurmountable problem.

Donaldson’s case was different from most people who undergo cryonics procedures, to the extent that he was requesting to accelerate his own death so as to make the cryonic preservation more efficient, as the deterioration of the brain (due to the growing tumor) would be stopped on time. As one legal scholar explained at the time, “doctors believe that if Donaldson waits until his natural death to be suspended, future reanimation will be futile because the tumor will have destroyed his brain” [35].

The doctors petitioned the courts an injunction against prosecution if they participated in inducing Donaldson’s death, for in normal circumstances, that would amount to murder. The doctors appealed to previous jurisprudence in which patients were granted the right to have life-saving treatment removed. But Donaldson’s case was different, because he was requesting active participation in hastening his death (i.e., active instead of passive euthanasia). Donald’s request was denied by the courts, he appealed to the California Court of Appeals, and it was likewise dismissed.

Donaldson therefore did not undergo euthanasia. In fact, he surpassed the physicians’ expectations, and he survived until 2006. His case presents interesting bioethical questions. Was Donaldson’s request any different from other patients who have requested euthanasia? Is hastening the death of a patient in order to stop the deterioration of the body so as to improve cryopreservation a form of euthanasia? In what follows, we will attempt to answer these questions. To do so, we will consider the doctrine of double effect and the definition of death. We will make the case that the current definition of death may not be entirely accurate. If an alternate definition of death is allowed, then Donaldson’s request was not necessarily about euthanasia, but about something else. If Donaldson was not requesting to be killed (even though the procedure may have ultimately killed him), then under the doctrine of double effect, the procedure would be morally acceptable.

## What is death?

Euthanasia seems a very straight-forward phenomenon. Etymologically, it means “good death”, and it can be formally defined as “the hastening of death of a patient to prevent further sufferings” [2]. But defining death is not as straight-forward as defining euthanasia.

Historically, the concept of death has been quite fluid. The phenomenon of premature burial was not unheard of in previous epochs. It caught the attention of one physician, Franz Hartmann, who reported in 1896 seven hundred cases [18]. He undertook some exhumations and found evidence of premature burial in nearly two per cent of them. Similarly, in 1905 William Tebb found evidence of 149 cases of live burial [50]. In the 19^th^ Century, this was a terrifying prospect in public imagination, so much that Edgar Allan Poe famously wrote the horror story *The Premature Burial*, and some people currently suffer from taphophobia, the fear of being buried alive as a result of an incorrect determination of death [7]. This suggests that the definition of death was rather sloppy, as in many cases, people were incorrectly pronounced dead, presumably because physicians were not following the proper signs.

The concern with premature burial elicited some initial interest in reforming definitions of death, but technological advances eventually became the motivational drive. As artificial respirators became available in the 1940s, new ways of defining death were considered. Before the advent of respirators and other equipment in intensive care units, it was observed that neurological, cardiac and respiratory functions were all intertwined. Consequently, it was assumed that when one system failed, the rest also failed.

This assumption is no longer tenable, because medical advances in life-support technology prove that some people can endure cardiac and pulmonary functions for long periods after the brain has ceased working. For example, there are many cases of pregnant women with no brain activity, in which they can still be kept in life-support systems for weeks, until the fetus is apt for delivery. In one study with 30 cases of pregnant women with no brain activity, 12 infants were born and survived. It was found that the mean gestational age at the time of brain death was 22 weeks, and mean gestational age at delivery was 29.5 weeks, meaning that those women survived at a mean of 7.5 weeks after brain death [10].

Other more extreme cases have been reported. For example, Shewmon reports the case of a 22-year-old man who had no brain activity, and has continued cardiopulmonary function for 18 years [41]. Cases of “locked-in syndrome” also complicate definitions. In these cases, patients are completely paralyzed, yet seemingly have consciousness (inferred from responses using eye movement as signals) [44].

The prevailing model for death is the Harvard criterion, which stipulates that death occurs when all three regions of the brain (cerebrum, cerebellum, and brain stem) stop functioning [55]. Physicians determine this with a series of test that seeks to establish the irreversibility: no response to stimuli, no respiration or spontaneous movement, no reflexes whatsoever, and perhaps most importantly, flat lines in the electroencephalogram [16], which indicate no electrical activity in the whole of the brain.

While this criterion seems robust now, some clinicians have expressed doubts. The ceasing of brain function does not guarantee that it is irreversible. Some clinicians have expressed concerns about the lack of certainty even with the conservative Harvard criterion. Bioethicist Robert Veatch concludes that “we are left with rather unsatisfying results. Most of the data do not quite show that persons meeting a given set of criteria have, in fact, irreversibly lost brain function. They show that patients lose heart function soon, or that they do not recover” [51].

We must not lose sight of the historical dimension. Definitions of death have adapted to emerging biomedical technologies. The Harvard criterion would not have been complete before the invention of the electroencephalogram. This suggests that we cannot be yet certain of what death is because there may very well be future technologies that force us to reconsider the definition, very much as the electroencephalogram forced us to abandon a definition under which, in the 19^th^ Century, people were buried alive.

Cryonics enthusiast Ralph Merkle makes the meaningful point that “current criteria are adequate to determine if current medical technology is likely to fail to revive a patient, but are silent on the capabilities of future medical technology” [26]. The technological feasibility of cryonics may still be at a very rudimentary level (and admittedly, it may never deliver its promises), but it at least forces us to reconsider the definition of death, given that future medical technologies may alter our current definition.

Proponents of cryonics offer an alternative definition of death, on the basis of the information-theoretic criterion. As per this definition, “if the structures in the brain that encode memory and personality have been so disrupted that it is no longer possible in principle to recover them, then the person is dead. If they are sufficiently intact that inference of the state of memory and personality are feasible in principle, and therefore restoration to an appropriate functional state is likewise feasible in principle, then the person is not dead” [27]. It is interesting to note that the information-theoretic criterion of death is typically only defended by enthusiasts of cryonics and is therefore seen as a somewhat fringe concept. However, upon closer examination, it becomes clear that this criterion overlaps with theories of personal identity that have been extensively defended by philosophers over various centuries, and that are widely discussed today. Perhaps the philosophers who uphold those theories of personal identity are timid in embracing the metaphysical foundations of cryonics, because in popular imagination cryonics remains an eccentric project more akin to sci-fi entertainment than serious academic discussion. But this is an unfair representation, for as it will be discussed below, the way cryonics’ enthusiasts think about death does deserve the attention of scholars.

The information-theoretic definition of death is complementary of the psychological criterion for personal identity. Evidence is not conclusive regarding what most people’s intuitions are when it comes to selecting personal identity criteria. On the one hand, extensive research has shown that people are intuitively dualists, and therefore accept that there is an immaterial substance that forms the core of a person’s identity [14, 17, 38, 46]. On the other hand, for mundane activities, most people use the body as a criterion of personal identity to navigate the world, and that is the basis for our daily recognizing of friends. Under this criterion, a person remains numerically the same, if and only if, he or she preserves the same body [31].

But a thought experiment developed by Sidney Shoemaker suggests that the body cannot be the definitive criterion for personal identity: “Two men, a Mr. Brown and a Mr. Robinson, had been operated on for brain tumors, and brain extractions had been performed on both of them. At the end of the operations, however, the assistant inadvertently put Brown’s brain in Robinson’s head, and Robinson’s brain in Brown’s head”. Who would be the person in Robinson’s body? Shoemaker argues that in this scenario, this person “recognizes Brown’s wife and family (whom Robinson had never met), and is able to describe in detail events in Brown’s life, always describing them as events in his own life. Of Robinson’s past life, he evidences no knowledge at all” [42]. Shoemaker firmly believes that the person would be Brown (even in Robinson’s body), suggesting that the brain is the truly relevant criterion for personal identity. It is presumably on the basis of this criterion that many proponents of cryonics posit that only preservation of the brain is necessary for survival [52].

But as per the information-theoretic criterion, it is not even preservation of the brain, but preservation of the memories, what really determines whether someone continues to be alive. Consequently, personal identity is determined, not by continuity of the body or the brain, but rather, by psychological continuity. A thought experiment by John Locke suggests that this is the case: “For should the soul of a prince, carrying with it the consciousness of the prince’s past life, enter and inform the body of a cobbler, as soon as deserted by his own soul, every one sees he would be the same person with the prince, accountable only for the prince’s actions: but who would say it was the same man?” [24]. Locke’s argument is that if one day, someone with the body of a cobbler wakes up with the thoughts of a prince (even with the brain of the cobbler), that person would be identical to the latter, not the former.

Consequently, the end of a person’s existence ought not to be defined by the end of particular physiological functions, but rather, by the annihilation of the very thing that defines them or serves as criterion of their personal identity: the information that psychologically constitutes them. The implication of this is that even if the patient does not have the physiological functions in conventional definitions of death, he/she may not be necessarily dead. If there is no brain activity, but tissue decay is stopped to the point that information is preserved, then the person is not dead.

Admittedly, this psychological criterion of personal identity is not without problems. It might be posited that downloading the autobiographical information of a person onto a computer does not guarantee the continuity of the person’s existence. Plenty of autobiographical information is found in St. Augustine’s *Confessions* and this book continues to be published, but that does not imply that Augustine (the person) continues to exist. Yet, this example would not be altogether challenging, because despite the extensive information contained in the *Confessions*, it is not a full reconstruction of Augustine’s biographical information, and the book by itself would not perform the typical features associated with intelligent and sentient beings as stipulated by Turing’s test [15].

Yet even if full autobiographical information is stored in a computer, and this information is then transferred into a living brain, would the person with that brain be identical to the one who originally produced those thoughts? For this case, intuitions vary. Some people (perhaps the majority) would think that the person would have the memories uploaded on the brain, but they would be *false* memories, and consequently, he or she would not be identical to the original person, since the new person did not really experience the things ‘remembered’. This has been amusingly explored by science fiction. For example, in the classic 1982 film *Blade Runner,* replicants are implanted with new memories, but the film’s storyline suggests that the replicants are not identical to the persons whose memories are being replicated.

The problem of false memory and how it relates to personal identity has also been addressed by philosophers. In a famous thought experiment, Bernard Williams imagines the case of a man named Charles: “[Suppose that] all the events he claims to have witnessed and all the actions he claims to have done point unanimously to the life-history of some one person in the past—for instance, Guy Fawkes. Not only do all Charles’ memory-claims that can be checked fit the pattern of Fawkes’ life as known to historians, but others that cannot be checked are plausible, provide explanations of unexplained facts, and so on. Are we to say that Charles is now Guy Fawkes, that Guy Fawkes has come to life again in Charles’ body, or some such thing?” [54]. Williams firmly establishes that no matter how much Charles may “remember” being Guy Fawkes, he is not Guy Fawkes. This suggests that the psychological criterion of personal identity is not adequate.

But then again, neither is the criterion based on the body or the brain. Yet another experiment (this time by Derek Parfit) suggests that brains and bodies cannot determine personal identity: “my body is fatally injured, as are the brains of my two brothers. My brain is divided, and each half is successfully transplanted into the body of one of my brothers. Each of the resulting people believes that he is me, seems to remember living my life, has my character, and is in every other way psychologically continuous with me. And he has a body that is very like mine” [33]. Theoretically, brains could undergo fission, and in that case, it is impossible to decide which of the two halves are numerically identical to the original one. Consequently, it appears that neither resulting half is identical to the original, which again suggests that personal identity cannot be enshrined in the brain.

Interestingly, very similar thought experiments have been used by some philosophers in order to uphold a soul criterion of personal identity. For example, Richard Swinburne [47] presents the scenario of Alexandra, a woman whose brain is split into two halves, and each half is transplanted to two new bodies resulting in two persons, Alex and Sandra. In Parfit’s presentation of the fission case, the implication is that neither of the resulting two persons can preserve the identity of the original person. But Swinburne suggests that assuming that fusion suddenly brings about the extinction of the original person is very implausible, and one (and only one) of the two resulting persons must be identical to the original person (even if we cannot know which one). In Swinburne’s view, this supports a soul criterion of personal identity: to the extent that there is an indivisible substance (i.e., the soul) within each person, fusion of the brain does not bring an end to the soul’s (and the person’s) existence. Swinburne therefore concludes that his “theory of personal identity does indeed lead to the theory that each human being consists of two substances, body and soul, and that it is our soul which makes each of us who we are.”

Swinburne’s argument is meaningful, but any defense of substance dualism must face a myriad of criticisms, and it is by no means clear that Swinburne (or any substance dualist) can overcome them. His position must face the problem of brain-mind correspondence, as well as interaction problems and the difficulty of incompatibility with our knowledge of the conservation of energy [6].

What, then, is the best criterion of personal identity? In light of all these difficulties, we posit that perhaps there is no criterion, and personal identity is an illusion. Robert Ettinger (the founder of the cryonics movement) came close to expressing that idea: “let us then cut the Gordian knot by recognizing that identity, like morality, is man-made and relative, rather than natural and absolute. Identity, like beauty, is partly in the eye of the beholder. It is only partly existent, and partly invented. Instead of having identity, we have degrees of identity, measured by some criteria suitable to the purpose” [11].

Ettinger’s idea is very similar to what is now called the “bundle theory of self”, as per the formulation offered by philosopher David Hume in the 18^th^ Century: “I may venture to affirm of the rest of mankind, that they are nothing but a bundle or collection of different perceptions, which succeed each other with inconceivable rapidity, and are in a perpetual flux and movement”. [20]. Realizing the difficulties of coming with a robust criterion for personal identity, Parfit upholds a similar view, arguing that in contemplating the prospects for survival, identity does not matter. What truly matters is the continuation of a person who is psychologically continuous with the present self. This in contrast to various other models, in which personal identity is assured, whether by appealing to immaterial substances such as the soul [48], or to the body [32]. In these models, personal identity does persist, given that either the soul or the body continues to exist. However, as argued above, these alternative models have difficulties of their own, and therefore it is more plausible to argue that personal identity does not truly persist.

This has implications for cryonics, and any prospect of radical life extension and immortality as a whole. Apart from cryonics, transhumanists have contemplated the prospect of mind uploading [53]. The resulting person may not even have an organic body, but that does not necessarily imply a failure of survival. As long as the information of the psychological contents from the original person have been preserved, survival is still a possibility.

As admitted above, the psychological criterion of personal identity is not without problems. And this implies that a seemingly unsurmountable problem in cryonics is whether the revived person is identical to the one whose brain was cryonically preserved. But defenders of the cryonics project can circumvent this objection if they emphasize the truly relevant goal of the cryonics project. If they make it clear from the outset that in survival, identity is not the ultimate concern, then they may be on safer grounds. In anticipating objections about the weak identity claim, they may very well echo Parfit’s clarification: “[in survival] *personal identity is not what matters*. I claim *What matters is Relation R:* psychological connectedness and/or continuity, with the right kind of cause… in an account of what matters, the right kind of cause could be any cause” [33].

This conclusion stems from the fact that, with any criterion of personal identity, there are difficulties. So, perhaps there is no assured way that personal identity can persist through time. Given that the preservation of identity may itself be hopeless endeavor, then cryonics should aim for survival, regardless of whether or not the revived person is identical to the person who lived before. Provided there is psychological continuity, the survival condition is satisfied, and that is what really matters. In contemplating the prospect of cryonics, it should not be disturbing that the revived person is not identical to the current person, in the same manner that, as per Hume’s bundle theory of the self, it is not disturbing that an adult person today is not identical to the child with the same DNA that was born some decades ago. If identity is not assured throughout someone’s lifetime, it is not assured in cryonics, either. But, once again, the crucial point is that it does not matter. Survival is what matters, regardless of whether the identity conditions are satisfied.

As far as enthusiasts of cryonics are concerned, there may be more than one revived person that displays psychological continuity with the original person. Suppose that during the cryonic revival process, duplication happens, and now, there are *two* revived persons with the same mental contents. They both think and feel continuous with the original person. As per the transitivity law of identity, these two revived persons cannot be both identical to the original person. This seemingly proves that neither one of the revived persons is identical to the original person. This would be irrelevant to the ultimate goal of cryonics. Cryonics seeks to ensure survival, and in the duplication scenario, the survival is accomplished, even if continuity of identity is not.

For cryonics, this implies that any hope of survival must ensure that the information encoded in the brain is rescued. And given that survival is determined by the psychological information, then the lack of brain activity does not necessarily imply the loss of such information. If the encephalogram’s line is flat, but the brain is preserved, then it may be that the information is still encoded in the brain, and such information may be retrieved at a later time.

There are some events after the ceasing of brain function, that would certainly imply death. Merkle claims that “an example of information-theoretic death is a person who suffers a heart attack and is cremated” [26]. In that case, the brain would turn to ashes, and it defies imagination to envision how the information originally encoded in the brain can be retrieved. But if the brain is cryonically preserved, it *is* possible to envision that the information may be retrieved at some later point, if biomedical technological advance continues.

An analogy with computers and their storage of information is called for. Suppose a computer stores important information that could solve the problem of world hunger, but due to some technical hardware problem, it ceases functioning, and no technician is able to repair it. What should we do with that computer? Given that the information cannot be accessed, one might think that the computer should simply be thrown away and torn to pieces, as it has ceased to function. But shall we value the computer on account of its current functioning, or on account of the information it holds?

Since the information is so vital, it would be foolish to simply throw away the computer. It is better to keep it in safe storage, as we come to acknowledge that it hosts important information, and we hope to figure out a future way of retrieving it. If that is ever accomplished, the computer would continue to exist. Francesca Minerva explains that “for all intents and purposes, it [the computer] would be equivalent in terms of information contained to the one I had, because all (or at least a large part of my files) would be retrieved and uploaded” [28]. The computer is defined by its information, and as long as its information remains safe (even though there is no known way of retrieving it), the computer has not ceased to exist. Likewise, a person’s survival may be defined by his or her psychological information, and if that information remains stored via cryopreservation of the brain (even if the brain is not functioning), one may argue that survival has been assured.

It is worth emphasizing (yet again) that there may be grounds for admitting that this computer analogy does not forcefully prove the continuation of personal identity in cryonics. A person’s mental contents may be saved on a computer and then transferred to another body, but it is not clear that the new person is identical to the original person. Nevertheless, as explained above, given the uncertainty of how personal identity is sustained, it is plausible to subscribe Parfit’s view that what matters in survival is psychological continuity, and not identity (for, continuity of identity is not guaranteed even within our own lifetimes).

Consider now Donaldson’s case. He seemingly requested the courts to allow physicians to hasten his death. But as per the above argumentation, that was not really the case. If we accept the information-theoretic criterion, he only asked to employ a procedure to halt the further deterioration of his brain, by inducing the ceasing of his neurological and cardiopulmonary functions, and cryonically preserving his brain. This would not amount to killing Donaldson, because the procedure would be done to preserve the information in his brain, and as long as that is achieved, Donaldson would not have ceased to exist, just as the nonfunctioning computer that hosts the important information would still continue to exist.

If the courts granted Donaldson’s request, he would not have undergone euthanasia, but rather, something that can be called “cryocide”. Ole Martin has coined this term, and in his view, “some of the central ethical arguments against euthanasia or assisted suicide would not apply to cryocide, for the aim of cryocide would not be to end life, but to preserve it” [30]. Other authors have used other terms, but the argument remains the same. For example, Minerva and Sandberg term this prospect “cryothanasia”, but likewise believe that “classical objections to euthanasia, based on the principle that it is always morally wrong to kill an innocent person, cannot be used to oppose cryothanasia” [29].

The central difference between euthanasia and cryocide is that in the former, a person is killed, whereas in the latter, a person is not. Now, it may very well be that cryonics never delivers its promises, and in that case, the information from cryonically preserved brains is never retrieved, and patients do die in the information-theoretic sense. In that case, conscious living time was taken away from the person who underwent cryocide, and this is a harm. Would cryocide then be as ethically objectionable as euthanasia? Let us now consider the doctrine of double effect to answer this question.

## The doctrine of double effect

Euthanasia is ethically opposed on many grounds, but a central one is that it violates the principle of double effect. Actions that result in the death of someone are not necessarily immoral, but as per this doctrine, certain requirements must be met. This doctrine posits that on occasion, it is morally acceptable to cause a harm if it comes as a side effect of bringing about a good result (hence, the double effect), provided the harm is not used as a means to bring the good result [5].

The origins of this doctrine can be traced to Medieval discussions on self-defense. Is it ever acceptable to kill someone in self-defense? Augustine did not think so, because “private self-defense can only proceed from some degree of inordinate self-love or ‘wrong-headed’ desire. In defending himself a man’s egoism either manifests or gains control over his action, and the passion of selfishness, concupiscence or libido warps his moral judgement so far as to render him totally incapable of deciding rightly between himself and his neighbor” [37].

But Thomas Aquinas countered that “nothing hinders one act from having two effects, only one of which is intended, while the other is beside the intention. … Accordingly, the act of self-defense may have two effects: one, the saving of one’s life; the other, the slaying of the aggressor” [3]. Self-defense is an action that seeks to preserve one’s life; in that process, the aggressor may die, but that is never the intention, although it may be foreseeable.

In a medical context, this principle makes euthanasia morally impermissible. In euthanasia, the goal may be to relieve the patient’s pain, but it is done by inducing a permanent harm. Death is not merely a foreseeable but unintended side effect of the action; it is the very means to achieve the original goal.

Consider two cases frequently discussed in this context. First, a physician wishes to relief the intense pain of a patient, and consequently injects a large dose of morphine that results in death. In this case, the physician’s actions have resulted in the patient’s death, but the physician has not intended the patient’s death, and death has not been used as means for pain relief.

Second, a physician wishes to relief the intense of a patient but plans to do so by “mercy killing” the patient with morphine. This case would be an example of euthanasia. Under the principle of double effect, it is not morally permissible, because death has been intended as a means for pain relief.

When it comes to medical actions that result in the death of a patient, ethicist Daniel Sumasy has come up with a meaningful question that should be asked: “if the patient were not to die after my actions, would I feel that I had failed to accomplish what I had set out to do?” [4]. In the first case, the answer is “no”, in the second case, the answer is “yes”. Only those actions with a “no” answer are morally authorized as per the doctrine of double effect.

The doctrine of double effect must of course still face some criticism. Intention is a major aspect of moral permissibility in this doctrine, but some philosophers reject its relevance. For example, Peter Singer argues that “we cannot avoid responsibility simply by directing our intention to one effect rather than another. If we foresee both effects, we must take responsibility for the foreseen effects of what we do” [43]. As per Singer’s consequentialist reasoning, we ought to give greater moral weight to the outcomes of actions. Simply stating that a particular harmful event was foreseen but not intended is an insufficient excuse.

Some criticism has also been levelled at the doctrine’s insistence on not actively doing deliberate harm. This is important in the context of euthanasia. Consider a patient who is offered life support, and expressly requests to have the equipment removed and let him/her die. This case may be called “passive euthanasia”, but it is not ethically objectionable on account of the doctrine of double effect. No action is being taken to kill the patient, at most, the patient dies from *inaction.*

Some philosophers reject any meaningful moral difference between killing and letting die. For example, James Rachels considers two cases. First, a woman wants her uncle dead, and serves poison in his coffee. Second, a woman wants her uncle dead and observes that the man drinks poison from somewhere else, and consequently decides not to give him the antidote [36]. The first case is about killing, the second one is about letting die, but according to Rachels, there is no moral difference. If in this case killing and letting die are both morally objectionable, in euthanasia, killing and letting die must both be equally objectionable (or commendable). Consequently, Rachels rejects the doctrine of double effect, and he uses this stance to uphold the moral permissibility of euthanasia.

While these philosophical musings can be expanded significantly, we posit that our intuitions strongly support the doctrine of double effect, and despite criticisms, the doctrine remains favored by both philosophers and the lay public. This has been tested with trolley dilemmas [1]. In the first scenario, a trolley is going on the tracks, and ahead there are five people tied and unable to move; should a lever be pulled so as to divert the trolley to another track in which one person is tied? About 90% of people respond that, yes, the lever should be pulled [8]. In the second scenario, a trolley is going down the tracks, and ahead there are five people tied and unable to move; this time, however, the trolley is about to go under a bridge, and standing on that bridge is a fat man. If the fat man is pushed to the tracks, his weight can stop the trolley; he would die, but the five tied people would be saved. Should the fat man be pushed? In this case, surveys show that approval is much lower [49]. In the first case, the doctrine of double effect authorizes pulling the lever; in the second case, the doctrine does not authorize pushing the fat man. The moral intuitions of most people coincide with this judgment.

For now, then, we may accept the doctrine of double effect, and consequently, condemn euthanasia. But can cryocide also be criticized on the grounds of this doctrine? Let us now consider each of the conditions for an action to be morally acceptable under the doctrine of double effect. To do so, we must keep in mind the proviso of accepting the information-theoretic criterion of death.

In its modern variant, Mangan describes the doctrine of double effect as follows: “a person may licitly perform an action that he foresees will produce a good and a bad effect provided that four conditions are verified at one and the same time: 1) that the action in itself from its very object be good or at least indifferent; 2) that the good effect and not the evil effect be intended; 3) that the good effect be not produced by means of the evil effect; 4) that there be a proportionately grave reason for permitting the evil effect” [25].

Is cryocide a good or indifferent action? It is difficult to decide this point. Cryocide would be the ceasing of cardiopulmonary and brain function, but with the expectation that it will be restored at a later time. In this regard, it is not substantially different from, say, anesthesia or induced coma. In both these cases, the patient may be deprived of consciousness, but this is a morally indifferent action, especially considering that it has a moral purpose. Cryocide, anesthesia and induced coma may lead to death, but they are not evil by and of themselves. This is different from euthanasia, in which the action is not good or indifferent. In euthanasia, the patient is actively killed, and this is evil. However, as it will be discussed below, the current risks of cryocide may be sufficiently large so as to admit that cryocide is not merely indifferent, but rather, evil.

In cryocide, what effects are intended? Clearly, the intention is not to kill the patient, but rather, to preserve the brain so as to avoid death. Ultimately, that may fail, and the patient may die, but that is not intended. Recall Daniel Sumasy’s question: “if the patient were not to die after my actions, would I feel that I had failed to accomplish what I had set out to do”. In cryocide, the answer is “no”. In fact, it is the opposite: the sense of accomplishment comes from the patient *not* dying after the cryonically preserved brain is restored, and the patient goes on living. This answer to Sumasy’s question provides another important moral distinction between euthanasia and cryocide. In euthanasia, if the patient does not die, there is no sense of accomplishment.

Does cryocide seek to produce the good effect by means of the evil effect? Again, no. If the patient dies, that is an evil effect. But even in that scenario, that would not be a means to reach the desired effect, i.e., repair of brain tissue. This is different from euthanasia. In euthanasia, the desired effect is the relief of pain. But death *is* the means to do so. If killing the patient fails in euthanasia, its goal has not been achieved. This implies that the bad effect (killing) is a means to the good effect (pain relief). In cryocide, the means-end structure is different. There may be death as bad effect, but it is never conceived of as a means to the good effect.

In cryocide, is there a proportionately good reason for permitting the bad effect? It is much more difficult to assess this condition. The good effect is the stopping of brain deterioration and eventual full restoration of brain function. This is clearly sufficiently desirable. But the assessment is complicated by the risks involved. Given that currently cryonics has no guarantee of success, the large risk factor gives reason to think that, as of now, cryocide is not simply morally indifferent,

Admittedly, cryonics is currently very risky, as there is no guarantee of its success. Given the high risk, it cannot be stated that, as of now, cryocide is simply morally indifferent. In order to preserve its moral quality, cryocide must have some considerable probability of success. At present, this requirement is not met. Until we have more robust evidence that cryocide would work (e.g., successful animal trials), a precautionary principle would be advisable.

Consider anesthesia for surgery. Is the good effect of temporary loss of sensation worth the risk of dying as a result of an overdose? Yes, because it removes pain during surgery and avoids shock, and the probability of dying while under anesthesia is much lower. But with cryonics, we do not know the risks. At present, there is no clear technological path to restoring brain function in cryonically preserved patients, so it seems that cryocide would inevitably result in death.

Cryonics has been criticized for its unrealistic demeanor, ultimately being described as sci-fi or religious fantasies, rather than real science. During preservation, tissue may be significantly damaged, given that organs undergo vitrification, and are at greater risk of fracture [13]. Likewise, ice crystals are likely to form, and this impedes adequate cell connection, thus making organ function far more difficult [12]. After restoration of physiological function, the body would also be affected by lack of oxygen, and this would in turn require regeneration technology that we currently do not possess.

Given the low probability of success in achieving the good effect, it would not seem certain that in cryocide there is an adequate proportion between the probability of obtaining the good effect, and the probability of obtaining the bad effect.

But all of this remains speculative. Recall that criteria of death has been adjusted to technological changes and given the accelerated pace of technological innovations in the last century, we may project that eventually, scientists will figure out how to restore brain function in cryonically preserved patients. For example, futurist Ray Kurzweil argues that “change of our human-created technology is accelerating and its powers are expanding at an exponential pace. Exponential growth is deceptive. It starts out almost imperceptibly and then explodes with unexpected fury” [22]. If Kuzweil is correct, then even though admittedly at present there is no plausible way of restoring brain function in cryonically preserved patients, things may change in the not-too-distant future.

The idea that someone could be revived with defibrillators would have been laughable to people in the Middle Ages, and yet, today it is a common occurrence. Is it not possible that we may be under the same bias regarding what future technologies can achieve? We may be under the same bias towards cryonics as Medieval folks would have been towards defibrillators.

Furthermore, Minerva points out that in cryonics, “the treatment is not futile, but experimental” [28]. We simply do not know yet how likely it is to succeed, because there is neither evidence in favor nor against its workability.

In any case, even if the probability of success is very low, is cryocide truly not proportional to the bad effect that it may bring about? This must be decided on a case-by-case basis. A 20-year-old person in good health has much to lose with the prospect of cryocide, because he/she would be unnecessarily risking his/her life, only to make a wild wager. But what about a terminal patient diagnosed with brain cancer? How much does this patient have to lose with cryocide? Such a person may not have much time to live anyways, so the risk may very well be worth it.

This decision could be based on what David Shaw calls the “cryonic wager”, on the model of Pascal’s argument in favor of believing in the existence of God. Pascal believed that in deciding to believe in God, there is much to gain and little to lose, for if God exists, the believer will have the infinite gain of heaven, whereas if God does not exist, the believer will only have minor finite losses [34]. A not insignificant number of people favor Pascal’s argument [39], and Shaw makes the case that if a similar wager is made in cryonics, it would be far more reasonable: “in the case of cryonics, it hardly seems fair to say the risk is so small; in the Cryonic Wager, we are merely being asked to hope that science might advance enough in the next few hundred years to permit safe thawing of frozen bodies, which sounds quite plausible when compared with believing in the eternal existence of an omnipotent deity” [40].

Now, Shaw is referring to cryonics, not properly cryocide. He argues that it is worth participating in the cryonics project after natural clinical death (as per the Harvard criterion). This is different from cryocide, in which clinical death is hastened. But we posit that the wager still holds, for a terminally ill patient may have little to lose.

Finally, it may be objected that, apart from the scientific risks entailed by cryocide, there is a metaphysical risk. This risk emanates from the fact that, given our certainty about the continuity of personal identity in the cryonically restored person, the very purpose of cryonics would be defeated. As discussed above, this is indeed a huge challenge to cryonics. But a plausible approach to this problem is to simply bite the bullet and admit that, indeed, the preservation of personal identity is not guaranteed in cryonics. However, with this admission, it must also be emphasized that personal identity is not the unified concept we are accustomed to, but rather, the self is a bundle or collection of different perceptions (in Hume’s words). This implies that continuity of personal identity is not even guaranteed within someone’s lifetime: an 80-year-old person today is not the same person as the child who, seventy years ago, had the same DNA and was called by the same name. If we can accept this insight, then it should not be particularly troubling that the cryonically restored person is not numerically identical to the original person. Under this conception, cryonics may not guarantee continuity of personal identity, but it does offer survival.

## Conclusion

Cryonics is seldom taken seriously, because of its alleged poor technological feasibility. But if the history of technology is any guide, such conclusions may be premature, and the jury is still out whether cryonics is hype or hope.

One infrequently discussed ethical aspect of cryonics is its potential to work around the objections typically levelled against euthanasia. Perhaps the central criticism against euthanasia appeals to the doctrine of double effect. If the information-theoretic criterion of death is accepted, then cryocide is in good position to avoid this criticism, given that in this prospect, the ceasing of physiological functions would be hastened and that may ultimately lead to death, but the bad effect would not be intended, and it is not framed as a means to achieve the end.

Of course, this possibility would force us to reconsider many other things. How would insurance companies deal with their business model once death is defined with the information-theoretic criterion? [45] How would society ensure that cryonics (a quite expensive procedure at present) does not generate massive inequality? [19].

Perhaps more importantly, there is no clearcut ethical answer to a question raised at the introduction of this article: should the California courts have given Donaldson’s physicians an injunction against prosecution if they participated in inducing Donaldson’s death (as per the Harvard criterion)?

These questions must be answered in the coming years, and that can only be done by engaging with the issue of cryonics (and especially its relationship to bioethics), rather than dismissing it simply as a silly sci-fi topic.

## Data Availability

Not applicable.

## References

[1] Andrade G. Medical ethics and the trolley problem. J Med Ethics Hist Med. 2019; 12:3. PMC6642460 31346396

[2] Annadurai K, Danasekaran R, Mani G (2014). Euthanasia: right to die with dignity. J Family Med Primary Care.

[3] Aquinas T. Summa theologica. New York: Xist Publishing; 2015.

[4] Bass M. Palliative care resuscitation. New Jersey: Wiley; 2006.

[5] Boyle JM (1980). Toward understanding the principle of double effect. Ethics.

[6] Calef S. Dualism and mind. The internet encyclopedia of philosophy. 2005.

[7] Cascella M (2016). Taphophobia and ‘life preserving coffins’ in the nineteenth century. Hist Psychiatry.

[8] Cloud J. Would You Kill One Person to Save Five? New Research on a Classic Debate. Time. 2011. https://healthland.time.com/2011/12/05/would-you-kill-one-person-to-save-five-new-research-on-a-classic-debate/. Accessed 15 Dec 2022.

[9] Emanuel EJ, Onwuteaka-Philipsen BD, Urwin JW, Cohen J (2016). Attitudes and practices of euthanasia and physician-assisted suicide in the United States, Canada, and Europe. JAMA.

[10] Esmaeilzadeh M, Dictus C, Kayvanpour E, Sedaghat-Hamedani F, Eichbaum M, Hofer S, Engelmann G, Fonouni H, Golriz M, Schmidt J (2010). One life ends, another begins: Management of a brain-dead pregnant mother-A systematic review. BMC Med.

[11] Ettinger RC, Rostand J. The prospect of immortality. New York: Doubleday New York; 1964.

[12] Fahy GM, Levy D, Ali S (1987). Some emerging principles underlying the physical properties, biological actions, and utility of vitrification solutions. Cryobiology.

[13] Fahy GM, Saur J, Williams RJ (1990). Physical problems with the vitrification of large biological systems. Cryobiology.

[14] Fiala B, Arico A, Nichols S. On the psychological origins of dualism: Dual-process cognition and the explanatory gap. Creating Consilience: Integrating the Sciences and the Humanities. 2012; 88–110.

[15] French RM (2000). The Turing Test: the first 50 years. Trends Cogn Sci.

[16] Giacomini M (1997). A change of heart and a change of mind? Technology and the redefinition of death in 1968. Soc Sci Med.

[17] Gut A, Lambert A, Gorbaniuk O, Mirski R (2021). Folk Beliefs about soul and mind: cross-cultural comparison of folk intuitions about the ontology of the person. J Cogn Cult.

[18] Hartmann F. Premature burial. Sonnenschein; 1896.

[19] Hughes J. The future of death: Cryonics and the telos of liberal individualism. J Evol Technol. 2001; 6.

[20] Hume D. A treatise on human nature: being an attempt to introduce the experimental method of reasoning into moral subjects, and dialogues concerning natural religion. longmans, green, and company; 1878.

[21] Joshi A (2016). A review and application of cryoprotectant: The science of cryonics. PharmaTutor.

[22] Kurzweil R. The singularity is near: When humans transcend biology. London: Penguin; 2005.

[23] LaBouff JP. He Wants to do what-cryonics: issues in questionable medicine and self-determination. Santa Clara Comput High Technol Law J. 1992; 8: 469. 11654757

[24] Locke J. An essay concerning human understanding. London: Kay & Troutman; 1847.

[25] Mangan JT (1949). An historical analysis of the principle of double effect. Theol Stud.

[26] Merkle R. Information-Theoretic Death. (n.d.). URL https://www.merkle.com/definitions/infodeath.html (accessed 6.9.22).

[27] Merkle RC (1992). The technical feasibility of cryonics. Med Hypotheses.

[28] Minerva F (2018). The Ethics of Cryonics: Is it Immoral to be Immortal?.

[29] Minerva F, Sandberg A (2017). Euthanasia and cryothanasia. Bioethics.

[30] Moen OM (2015). The case for cryonics. J Med Ethics.

[31] Noonan HW. Personal identity. New York: Routledge; 2019.

[32] Olson ET (2004). Animalism and the corpse problem. Australas J Philos.

[33] Parfit D. Reasons and persons. Oxford: OUP Oxford; 1984.

[34] Pascal B. Pensées and other writings. Oxford: Oxford University Press; 1999.

[35] Pommer RW III. Donaldson v. Van de Kamp: Cryonics, assisted suicide, and the challenges of medical science. J Contemp Health Law Policy. 1993; 9: 589. 10183890

[36] Rachels J. Killing and Letting Die. Encyclopedia of Ethics; 2001.

[37] Ramsey P. Basic christian ethics. Louisville: Westminster John Knox Press; 1950.

[38] Roazzi M, Nyhof M, Johnson C (2013). Mind, soul and spirit: Conceptions of immaterial identity in different cultures. International Journal for the Psychology of Religion.

[39] Rota M. Taking Pascal’s Wager: Faith. Evidence and the Abundant Life. Westmont: InterVarsity Press; 2016.

[40] Shaw D (2009). Cryoethics: seeking life after death. Bioethics.

[41] Shewmon D. Seeing is believing: videos of life 13 years after ‘brain death’, and consciousness despite congenital absence of cortex: 3rd International Symposium on Coma and Death. Havana, Cuba. 2000; 22–25.

[42] Shoemaker SS. Self-knowledge and Self-identity. Ithaca: Cornell University; 1958.

[43] Singer P. Practical ethics. Cambridge: Cambridge University Press; 2011.

[44] Smith E, Delargy M. Locked-in syndrome BMJ. 2005;330:406–9. 10.1136/bmj.330.7488.406 PMC549115 15718541

[45] Spector DR. Legal implications of cryonics. Clev.-Marshall L. Rev. 1969; 18: 341.

[46] Steven H. Whose Intuitions? Which Dualism? 1, in: The Roots of Religion. Routledge, pp. 37–54; 2016.

[47] Swinburne R. Are we bodies or souls? Oxford: Oxford University Press; 2019.

[48] Swinburne R (1984). Personal identity: The dualist theory. Personal identity.

[49] Tannsjo, T. Taking life: Three theories on the ethics of killing. Oxford: Oxford University Press; 2015.

[50] Tebb W, Vollum EP. Premature burial and how it may be prevented: with special reference to trance, catalepsy, and other forms of suspended animation. S. Sonnenschein & Company, lim; 1896 .

[51] Veatch R. Death, dying, and the biological revolution. New Haven: Yale University Press; 1989.

[52] Von Verschuer F. Freezing lives, preserving humanism: cryonics and the promise of Dezoefication. Distinktion J Soc Theory. 2020; 21: 143–161.

[53] Wiley K. A taxonomy and metaphysics of mind-uploading. Seattle: Humanity+ Press and Alautun Press; 2014.

[54] Williams BA. Personal identity and individuation. JSTOR. 1956; pp. 229–252.

[55] Youngner SJ, Arnold RM (2001). Philosophical debates about the definition of death: who cares?. J Med Philos.

